# Serum neutrophil gelatinase-associated lipocalin (NGAL) as a diagnostic tool in pediatric acute appendicitis: a prospective validation study

**DOI:** 10.1007/s00383-022-05197-w

**Published:** 2022-08-16

**Authors:** Javier Arredondo Montero, Giuseppa Antona, Carlos Bardají Pascual, Mónica Bronte Anaut, Raquel Ros Briones, Amaya Fernández-Celis, Adriana Rivero Marcotegui, Natalia López-Andrés, Nerea Martín-Calvo

**Affiliations:** 1 Pediatric Surgery Department, Hospital Universitario de Navarra, Calle Irunlarrea 3, 31008 Pamplona, Navarra Spain; 2 Pathology Department, Hospital Universitario de Araba, Vitoria, Spain; 3 Cardiovascular Translational Research, NavarraBiomed (Miguel Servet Foundation), Hospital Universitario de Navarra, Pamplona, Spain; 4 Clinical Analysis Department, Hospital Universitario de Navarra, Pamplona, Spain; 5 Department of Preventive Medicine and Public Health, School of Medicine, University de Navarra, Pamplona, Spain; 6 IdiSNA, Instituto de Investigación Sanitaria de Navarra, Pamplona, Spain; 7 CIBER de Fisiopatología de la Obesidad y la Nutrición, Instituto de Salud Carlos III, Madrid, Spain

**Keywords:** NGAL, Lipocalin, Gelatinase, Neutrophil, 24p3, Lipocalin, Siderocalin, LCN2, Pediatric acute appendicitis, Diagnostic, Specificity, Sensitivity, ROC, AUC

## Abstract

**Introduction:**

NGAL has recently been studied as a biomarker in the diagnostic context of pediatric acute appendicitis (PAA), although existing series are scarce and have limited sample sizes.

**Materials and methods:**

A prospective observational study was designed to validate serum NGAL as a diagnostic tool in PAA. This study included 215 patients, divided into 3 groups: (1) patients undergoing major outpatient surgery (*n* = 63), (2) patients with non-surgical abdominal pain in whom a diagnosis of PAA was excluded (*n* = 53) and (3) patients with a confirmed diagnosis of PAA (*n* = 99). Patients in group 3 were divided into complicated or uncomplicated appendicitis. In 201 patients, a serum sample was obtained at the time of diagnosis and NGAL concentration was determined by ELISA. The Kolmogorov–Smirnov test was used to assess normality. Comparative statistical analyses were performed using the Mann–Whitney *U* test, the Kruskal-Wallis test and the Fisher’s exact test. To calculate the discriminative ability of the molecule, the area under the receiver-operating characteristic curves (AUC) was calculated. A *p* value < 0.05 established statistical significance.

**Results:**

Median (interquartile range) of serum NGAL values were 38.88 (27.15–48.04) ng/mL (group 1), 51.84 (37.33–69.80) ng/mL (group 2) and 65.06 (50.50–86.60) ng/mL (group 3). The AUC (group 2 vs 3) was 0.642 (95% CI 0.542–0.741) (*p* < 0.001) and the best cutoff point was found to be at 40.97 ng/mL, with a sensitivity of 89% and a specificity of 34.6%. No statistically significant differences in serum NGAL values were found between patients with uncomplicated PAA and those with complicated PAA.

**Conclusions:**

This prospective validation study with a large sample size confirms that the diagnostic yield of NGAL in the context of PAA is only moderate, and therefore, it should not be used as a unique diagnostic tool. Furthermore, NGAL is not a valid biomarker to discern between uncomplicated and complicated PAA.

**Supplementary Information:**

The online version contains supplementary material available at 10.1007/s00383-022-05197-w.

## Introduction

The diagnosis of pediatric acute appendicitis (PAA) remains a challenge in Pediatric Emergency departments. The diagnosis of PAA is based on the anamnesis, physical examination and the presence of analytical alterations in the blood count and basic biochemistry, with leukocytosis, neutrophilia and elevation of acute phase reactants (such as CRP) being the most important. The diagnostic yield attributable to these analytical alterations is variable according to the series but is considered moderate [1]. The creation of ratios derived from these basic parameters, such as the neutrophil–lymphocyte index, has partially improved the diagnostic yield of these markers [2]. On the other hand, different novel biomarkers have recently been evaluated as potential diagnostic tools in PAA, with IL-6, PTX-3 and calprotectin standing out, although the performance of all of them seems to be only moderate [3–5].

In relation to radiological studies for the diagnosis of PAA, abdominal ultrasound (AUS) has an excellent diagnostic yield [6], especially when combined with clinical and analytical scores (such as PAS score or Alvarado score) [1]. However, AUS is an operator-dependent test and visualization of the appendix is not achieved in all patients, so it cannot be considered a definitive diagnostic test [6, 7]. It should be noted that the use of computed tomography (CT) in the setting of PAA is limited due to the significant radiation dose associated with this particularly vulnerable population. The overall diagnostic yield in relation to PAA varies according to series, with recent publications establishing a diagnostic error rate of 4% and a diagnostic delay rate of 63%, respectively [8, 9].

Serum neutrophil gelatinase-associated lipocalin (NGAL), also called lipocalin 2 (LCN2), 24p3 lipocalin or siderocalin is a small protein of 178 amino acids, which is expressed at low concentrations in various tissues of the human body, including the kidney, lung, and gastrointestinal tract. Although classically attributed with the function of sequestering siderophores as a bacteriostatic mechanism, it has recently been shown to be a useful marker in acute kidney injury [10].

In relation to gastrointestinal pathology, fecal NGAL has been evaluated as a potential marker in inflammatory bowel disease, with promising results [11]. Mucosal and fecal NGAL has also been evaluated in the context of collagenous colitis, and it has been shown that NGAL mRNA is upregulated in active disease and reduced in corticosteroid-induced clinical remissions, which is reflected in fecal NGAL concentrations [12]. Recent studies reported that NGAL could be useful in the diagnosis of pediatric acute appendicitis [13, 14]. However, those studies presented important flaws, such as small sample size [13] and the lack of a control group of patients with non-surgical abdominal pain [14], which might have hampered their results.

In this study, we aimed to assess the diagnostic performance of NGAL in PAA using a large cohort of children and accounting for the main limitations of previous studies.

## Materials and methods

This study was approved by our center’s clinical research ethics committee prior on December 18, 2020, under code PI_2020/112. The ethical principles of the Declaration of Helsinki were applied for the conduct of this research study. The parents or legal representatives of all participants signed an informed consent form prior to the inclusion in the study.

### Study design

This is a prospective observational study aimed to determine the diagnostic performance of serum NGAL in PAA. Three groups of pediatric patients were recruited for this study: (1) patients with no underlying pathology who came to undergo scheduled outpatient surgery, (2) patients with acute abdominal pain who attended the emergency department and in which acute appendicitis was finally excluded—also defined as non-surgical abdominal pain (NSAP), and (3) patients with histologically confirmed diagnosis of acute appendicitis (PAA group).

The purpose of including group 1 was to establish a baseline analytical range and to study the behavior of NGAL in a healthy pediatric population to serve as a comparison for the other two groups included in the study. To confirm that the patients in group 2 had not developed PAA on a deferred basis, a telephone call was made 2 weeks after inclusion in the study to confirm that they had not presented new symptomatology, nor had they consulted any other center for abdominal pain. In relation to the histopathological study, the same pathologist evaluated all the study specimens, with no knowledge of diagnostic suspicion in any of the patients. Patients in group 3 were stratified in relation to the histopathological study in uncomplicated PAA (congestive or phlegmonous/suppurative appendicitis) and complicated PAA (gangrenous or perforated appendicitis). Congestive appendicitis was considered polymorphonuclear infiltration of the appendix without invasion of the lamina propria. Phlegmonous or suppurative appendicitis was considered polymorphonuclear infiltration of the appendix with invasion of the lamina propria. Gangrenous appendicitis was considered polymorphonuclear infiltration of the appendix with invasion of the lamina propria and/or mural necrosis. Perforated appendicitis was considered to be any of the previous alterations with the presence of micro- or macroscopic perforation of the appendix. All the patients in group 3 were reviewed on an outpatient basis in the first postoperative month.

Patients were recruited when the personnel conducting the investigation were available at the center. The recruitment period extended from February to December 2021. Inclusion and exclusion criteria are shown in Supplementary file 1.

Information on sociodemographic and clinical variables were collected at baseline through a questionnaire completed by participant’s parents/legal tutor. Information on the surgical procedure, postoperative evolution, radiological findings, and histological analysis was extracted from participants’ clinical records by the principal investigator.

### Sample collection and measurement of serum NGAL

A venous blood sample was taken from each patient in a vacutainer tube with separator gel (3.5 mL). In patients in group 1, this sample was taken prior to the scheduled intervention. In patients in groups 2 and 3, it was taken at the time of inclusion in the study, during their stay in the emergency department. All the patients attended at our center were treated with the same antibiotic regimen, and in all of them, the antibiotic was administered after inclusion in the study and extraction of the analytical sample. No specific medication consumption that could alter the analytical results in any of the patients in the study was identified in the targeted anamnesis. Serum samples were frozen and processed by laboratory personnel blinded to the patient’s group. Serum determinations of NGAL were made using a commercial ELISA kit (Quantikine kit, R&D systems) and following the manufacturer’s instructions.

### Statistical analysis

Prior to carrying out the study, a calculation was made of the sample size required to achieve statistical significance. For this calculation, we relied on previous literature on the diagnostic performance of NGAL in PAA. Estimating an alpha value of 0.05 (two-sided) and a power of 0.8, a sample size of *n* = 16 per group was obtained for the study.

For descriptive purposes, the median and interquartile ranges were used for quantitative variables and proportions for categorical ones. Missing values in quantitative variables were imputed to the group-specific median. Kolmogorov–Smirnov test was used to assess the normality of quantitative variables. Sociodemographic and clinical variables were compared between the groups using the Kruskal–Wallis test, the Mann–Whitney *U* test and the Fisher’s exact test. To analyze the discriminative capacity of the NGAL, we calculated the area under the receiver-operating characteristic curves (ROC). In addition, the distance on the ROC curve of each NGAL value was calculated as the square root of [(1 − sensitivity)^2^ + (1 − specificity)^2^]. The NGAL value with the shortest distance on the ROC curve was considered in the determination of optimal cutoff. Alternative cutoff points were analyzed in terms of sensitivity, specificity, positive likelihood ratio and negative likelihood ratio. Pearson’s and Spearman’s correlation tests were performed to assess the relationship between NGAL and the sociodemographic variables of the study.

Statistical significance was settled in a *p* value < 0.05. Statistical analyses were performed with STATA 15.0 (StataCorp LCC).

## Results

### Demographic and clinical characteristics

Out of the 215 patients recruited, 14 (6.5%) were excluded from the analyses due to the lack of serum sample at the time of diagnosis (group 1 = 6 patients; group 2 = 1 patient; group 3 = 7 patients). One missing value in age was imputed to the group-specific median. Therefore, the final sample for this study included 201 participants divided into 3 groups: (1) patients who underwent major outpatient surgery (*n* = 57), (2) patients with NSAP in whom the diagnosis of PAA was excluded (*n* = 52) and (3) patients with a confirmed histopathological diagnosis of PAA (*n* = 92). Based on histopathological analysis, participants in group 3 were further subdivided into complicated PAA (*n* = 29) and uncomplicated PAA (*n* = 63). There were no negative appendectomies in our series. No other pathological findings in the cecal appendix of relevance were found in our series. Participants’ sociodemographic and clinical characteristics by group are shown in Table 1. The age range of the patients included in the study was 3–14 years. In relation to the sociodemographic characteristics of the series, children in group 1 were more likely male and slightly older. In relation to the clinical variables of groups 2 and 3, no statistically significant differences were found in the hours of evolution of pain, in the presence or absence of fever at home, in the number of diarrheal stools, in the presence of urinary symptoms or in the presence or absence of hyporexia. Significant differences were found, however, in the number of emetic episodes, total leukocyte count, total neutrophil count and serum C-reactive protein values (*p* < 0.001).

**Table 1:** Clinical characteristics and sociodemographic characteristics of the participants of the study

Clinical and sociodemographic variables	Group 1 (ambulatory controls) *N* = 57	Group 2 (NSAP) (*n* = 52)	Group 3 (PAA) (*n* = 92)	*p* value
Age (years)	8.62 (3.25)	11.09 (2.47)	9.61 (3.01)	< 0.001
Sex (male/female) (%)	46/11 (80.7%)	24/28 (46.15%)	59/33 (64.13%)	0.04
Hours of pain evolution		31.57 (23.12)	26.45 (18.90)	0.29
Fever > 37.8 (yes/no/missing data) (%)		15/37 (28.84)	29/62/1 (31.52)	0.85
Number of diarrheal stools		0.40 (1.20)	0.68 (2.48)	0.54
Urinary symptoms (yes/no) (%)		8/44 (15.38)	21/70/1 (22.82)	0.38
Number of emetic episodes		0.55 (1.96)	2.51 (2.47)	< 0.001
Hyporexia (yes/no/missing data) (%)		35/15/2 (70.58)	72/16/4 (78.2)	0.11
Total leucocyte count (1 × 10^9^/L)^a^		9.5 (7.8–12.5)	16.15 (13–19.1)	< 0.001
Total neutrophil count (1 × 10^9^/L)^a^		5.7 (4.1–8.4)	13.25 (9.7–16.5)	< 0.001
C-reactive protein (mg/L)^a^		1.55 (1–22.65)	28.15 (7.4–63.3)	< 0.001
Serum NGAL (ng/mL)^a^	38.88 (27.15–48.04)	51.84 (37.33–69.80)	65.06 (50.50–86.60)	< 0.001

Numbers are mean (standard deviation) or numbers (percentage)

^a^Median, IQR

### Serum NGAL

**Fig. 1:**
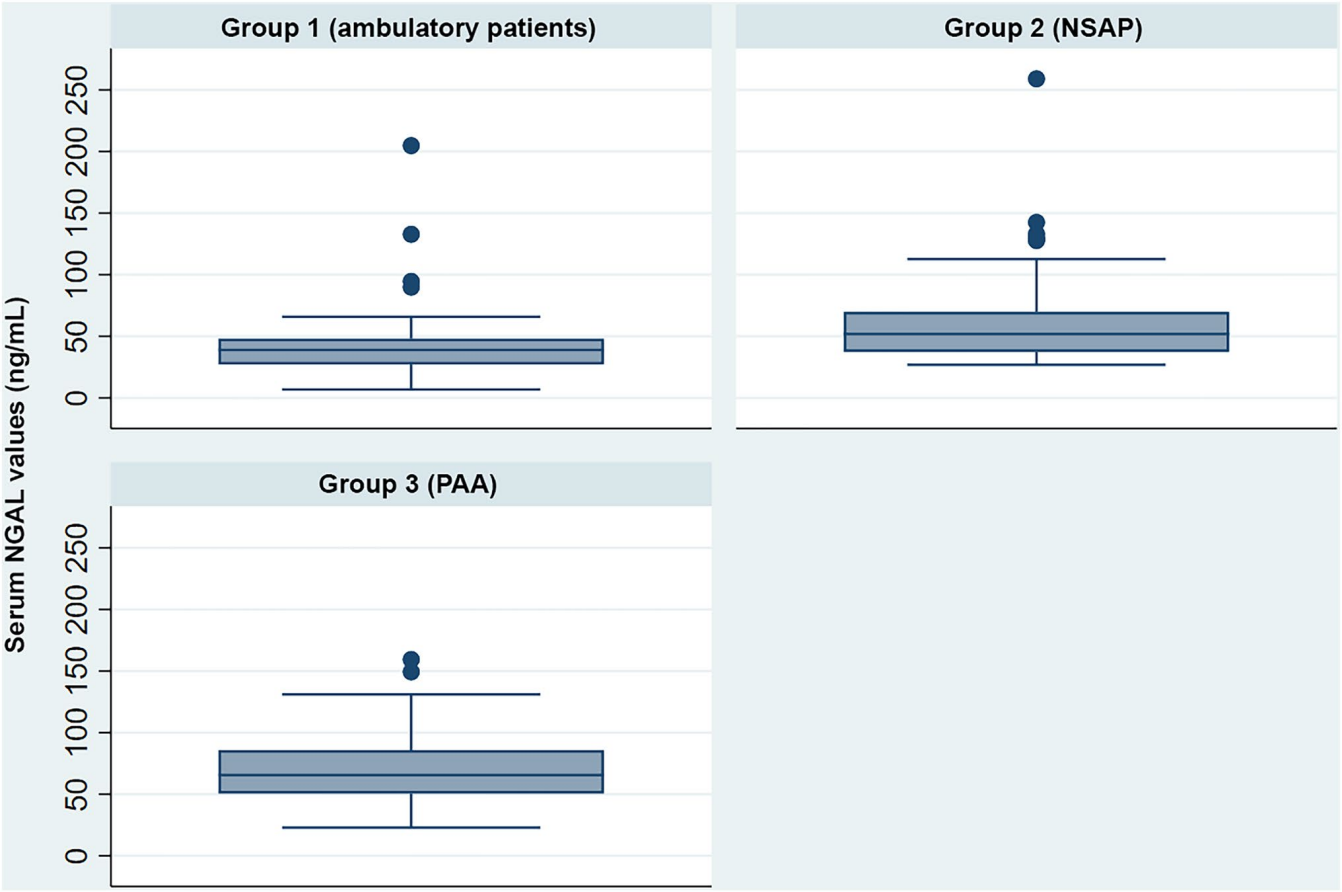
Box-plot representation of NGAL serum values in the different study groups

Median (IQR) serum NGAL values were 38.88 ng/mL (27.15–48.04) in group 1, 51.84 ng/mL (37.33–69.80) in group 2, and 65.06 ng/mL (50.50–86.60) in group 3 (*p* < 0.001). Median (IQR) serum NGAL values in non-complicated PAA were 64.99 ng/mL (50.52–86.64) and in complicated PAA were 66.09 ng/mL (50.50–82.82) (*p* = 0.64). The graphical representation of NGAL serum values by group is shown in Fig. 1.

**Fig. 2:**
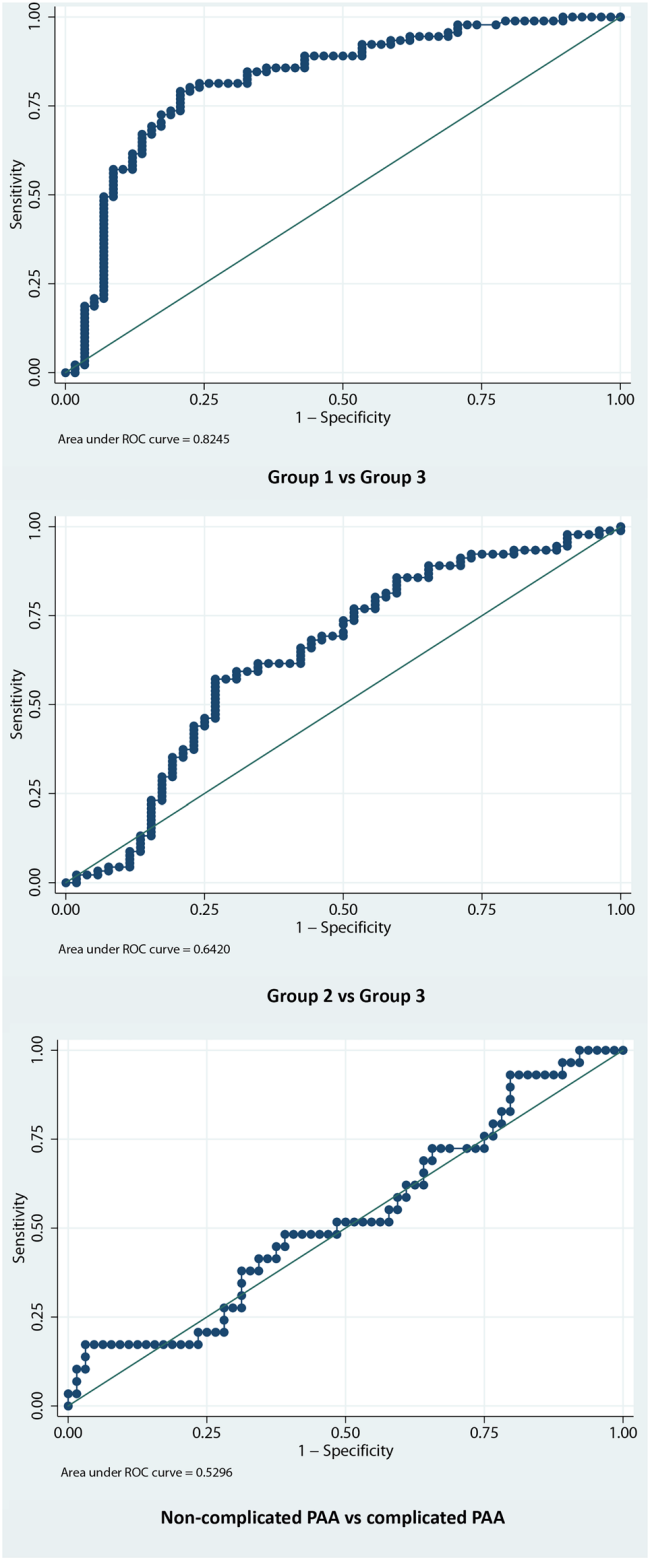
Graphical representation of the ROC curve regarding NGAL diagnostic performance. Above: Group 1 vs 3. Center: group 2 vs 3. Bottom: non-complicated PAA vs complicated PAA

Regarding the ability of NGAL to discriminate between patients in groups 2 vs. 3, we found an AUC of 0.64 (95% CI 0.54–0.74) (*p* < 0.001). The cutoff point with the shortest distance on the ROC curve was set at 40.97 ng/mL, with a sensitivity of 89% and a specificity of 34.6%. The AUC observed for the discrimination between patients in groups 1 vs. 3 was 0.82 (95% CI 0.75–0.90) (*p* = 0.04). In this analysis, the cutoff point was established at 54.92 ng/mL, resulting in a sensitivity of 68.1% and a specificity of 84.2%. We also analyzed the diagnostic performance of serum NGAL to distinguish between uncomplicated vs. complicated PAA and obtained an AUC of 0.53 (95% CI 0.40–0.66) (*p* = 0.37). For the cutoff point of 79.85 ng/mL, sensitivity and specificity were 37.93% and 68.75%, respectively. The graphical representation of the different ROC curves is shown in Fig. 2.

In addition, we calculated the sensitivity, specificity and positive likelihood ratio of alternative cutoff values for serum NGAL when trying to discriminate between patients with NSAP (group 2) vs. those with PAA (group 3). As expected, sensitivity decreased, and specificity increased as the cutoff point raised. The highest percentage of correctly classified participants was observed for the cutoffs 40.97 and 43.08 ng/mL. They both had a great sensitivity (89.01 and 85.71%, respectively) with a positive likelihood ratio of 1.36 and 1.43, respectively (Table 2).

**Table 2:** Proposed alternative cutoffs for serum NGAL (group 2 vs group 3)

NGAL cutoff value (ng/mL)	Positive likelihood ratio	Correctly classified (%)	Sensitivity (%)	Specificity (%)
22.91	1	63.64	100	0
**40.97**	**1.36**	**69.23**	**89.01**	**34.62**
43.08	1.43	69.23	85.71	30.38
60.66	2.12	62.94	59.34	69.23
102	0.89	39.16	13.19	86.54

Bold values indicate best cutoff

## Discussion

In the present study, we prospectively evaluated the diagnostic performance of serum NGAL in PAA using data of a cohort of 201 children. Our results show that the discriminatory capacity of NGAL to distinguish between NSAP and acute appendicitis is only moderate, and it is reduced to poor when it comes to distinguish between non-complicated PAA and complicated PAA.

As previous studies suggested [13], there is a biological plausibility that justifies the elevation of NGAL in the context of PAA. This molecule is secreted at the intestinal level and neutrophils are the first cells to be elevated in the context of an appendiceal infectious process, such as PAA. This would explain, at least partially, the poor diagnostic performance of neutrophil count in the context of evolved PAA.

The first study that evaluated the role of serum NGAL in the diagnosis of PAA consisted in a cohort of 60 pediatric patients [13]. Higher levels of serum NGAL were observed in the PAA group compared with either ambulatory controls or with the group of children with NSAP. The authors reported a sensitivity and specificity of 77.3% and 97.4%, respectively, when NGAL was compared with the Alvarado score when the cutoff point was set in 7, and a sensitivity and specificity of 100% and 55%, respectively, when the cutoff point was set in 9. Considering the results presented by the authors, it is striking that they have analyzed the diagnostic performance of NGAL with the Alvarado score instead of using, as a gold standard, the pathological anatomy that accurately demonstrates whether the patient had appendicitis or not. The authors concluded that although NGAL could be used as laboratory biomarker to discriminate between PAA and non-specifical abdominal pain, further studies, with large samples sizes, were needed to determine normal values of serum NGAL.

More recently, Kakar et al. published a similar study in a cohort of 92 patients [14]. They proposed that the cutoff point of serum NGAL should be established at 26.43 pg/mL, which resulted in a sensitivity and specificity of 68.3% and 65.5%, respectively. The authors reported significantly higher levels of serum NGAL in the group of patients with acute appendicitis, but, in this study, the control group consisted of patients recruited at the orthopedic department and without any abdominal symptom. As these authors did not include patients with NSAP, we believe that their results should be interpreted with caution. In that same study, the authors found that NGAL was not useful to assess the severity of the appendicitis, which is consistent with our results. Finally, Kakar et al. also reported that the diagnostic performance of urinary NGAL in PAA was poor, which motivated our choice of serum NGAL. It should also be considered that given the elevation of this marker in the context of renal damage, its use in the context of PAA may be compromised in patients with marked mucocutaneous dehydration or elevated creatinine at the time of diagnosis.

We believe that the use of the Alvarado score as gold standard [13] and the use of a control group of patients without abdominal symptoms [14] are important limitations that hamper the results of those studies in the assessment of the diagnostic performance of NGAL in PAA. Our results add to the previous knowledge because we used a rigorous definition of groups: cases of PAA were confirmed by histopathologic study and in controls PAA was reasonably ruled out after telephone contact 2 weeks after their visit to the emergency department. Furthermore, our control group consisted of children with NSAP because we believe that if NGAL were to be used in clinical practice, it would be to differentiate between patients with PAA and patients with NSAP.

The great disparity in the range of serum NGAL observed by these two working groups is noteworthy. Considering those results, we hypothesized that NGAL levels may be related to age, sex and body mass index. However, a correlation analysis of serum NGAL values and those variables resulted in very weak correlation coefficients (*r* = − 0.01 for age; rho = 0.12 for sex; and *r* = − 0.07 for BMI), which allowed us to rule out the possibility that between groups differences in NGAL serum may be due to residual confounding by sociodemographic variables. The great variability in the NGAL range could be due to intrinsic characteristics of the sample or to differences in the analytical procedure, but whatever the reason, that variability make it very difficult to infer a normal range of serum NGAL. Besides, it should be noted that since NGAL is not organ-specific, it should be considered with caution in patients with other pathologies, such as renal damage.

Our study has several strengths, such as the prospective design, the large sample size and the fact that the personnel who performed the analytical determinations were blinded to patient’s group. Furthermore, we used two different control groups, one of which consisted of ambulatory patients without abdominal pathology, which allowed us to define the normal analytical range of NGAL in our sample. The second control group consisted of patients with NSAP in whom there was clinical suspicion of PAA. From a clinical point of view, what is of greatest interest is to know the ability of NGAL to discriminate between patients with and without PAA in whom clinical inspection is insufficient and, therefore, we believe that using such a control group allows a better understanding of the potential of NGAL as a diagnostic tool.

Despite our findings, our study has some limitations. First, we used a convenience sampling, which may result in selection bias. Besides, 14 participants were excluded from the analyses because of the lack of serum sample. However, the strict adherence to the predefined inclusion and exclusion criteria makes such bias unlikely. Second, we cannot exclude the presence of unidentified confounding variables.

In conclusion, our results suggest that serum NGAL has a moderate discriminatory capacity to distinguish between PAA and NSAP, and a poor discriminatory capacity to distinguish between complicated and uncomplicated PAA. Therefore, our findings do not support the use of NGAL as a stand-alone tool in the diagnosis of PAA. The potential role of this biomarker as part of a score of risk that includes other clinical, analytical, and radiological variables should be analyzed in future studies. For this, it will first be necessary to know the reasons for the high variability observed between studies and to define a range of normal values.

## Supplementary Information

Below is the link to the electronic supplementary material.

Supplementary file1 (DOCX 16 KB)

## Data Availability

All data pertaining to this study are available upon justified request through the author in correspondence.
