# Supplementary material for: Serum neutrophil gelatinase-associated lipocalin (NGAL) as a diagnostic tool in pediatric acute appendicitis: a prospective validation study
Source: Pediatr Surg Int. 2022 Aug 16;38(11):1569–76. doi: 10.1007/s00383-022-05197-w (PMC9519728; doi:10.1007/s00383-022-05197-w)
Supplement: Supplementary file 1 — Supplementary file1 (DOCX 16 KB) [file 383_2022_5197_MOESM1_ESM.docx]

**Supplementary file 1. Inclusion and exclusion criteria**

**Inclusion criteria**

The study will include patients aged 0 to 14 years inclusive who present at the pediatric emergency department with acute abdominal pain suggestive of acute appendicitis (initially mesogastric pain and later radiating to the right iliac fossa, pain starting in the right iliac fossa) of less than 5 days of evolution and associated with at least one of the following symptoms: hyporexia, nausea, vomiting, febrile fever, fever, hyporexia, diarrhea. Inclusion in the group of cases will be confirmed with the anatomopathological diagnosis of the surgical specimen.

**Exclusion criteria**

- Patients with a clear suspicion of acute appendicitis or with clinical instability who do not require complementary tests prior to surgery.

-Patients with known renal pathology, marked mucocutaneous dehydration or elevated creatinine or urea in the diagnostic work-up

- Patients with metastatic neoplasia.

- Patients with hematological alterations.

- Patients with active autoimmune disorders.

- Patients previously appendectomized.

- Patients who have been treated with immunosuppressants in the 28 days prior to the evaluation in the ED.

- Patients who have been treated with systemic steroids in the 14 days prior to the emergency evaluation.

- Patients who have had abdominal trauma prior to ED evaluation.
